# Co-culture of human fibroblasts and *Borrelia burgdorferi* enhances collagen and growth factor mRNA

**DOI:** 10.1007/s00403-017-1797-1

**Published:** 2017-12-06

**Authors:** Elisabeth Aberer, Milana Surtov-Pudar, Daniel Wilfinger, Alexander Deutsch, Gerd Leitinger, Helmut Schaider

**Affiliations:** 1 Department of Dermatology, Medical University of Graz, Auenbrugger Platz 8, 8036 Graz, Austria; 2 Division of Haematology, Internal Medicine, Medical University of Graz, Graz, Austria; 3 Research Unit Electron-Microscopic Techniques, Institute of Cell Biology, Histology and Embryology, Medical University of Graz, Graz, Austria; 4 The University of Queensland Diamantina Institute, Translational Research Institute, University of Queensland, Brisbane, QLD Australia

**Keywords:** *B. burgdorferi*, *B. afzelii*, Fibroblast, Co-culture, mRNA type I collagen, TGF-β, FGF-1, PDGF, Decorin, Calreticulin, Fibrosis

## Abstract

Skin fibrosis has been reported in *Borrelia burgdorferi* infection in Europe, but has been questioned by several authors. The objective of the present study was to examine the interaction of skin fibroblasts with *B. burgdorferi* sensu stricto B31 (BB) and *B. afzelii* (BA) in vitro by electron microscopy. We also determined the expression of collagen type I, TGF-β, FGF-1, calreticulin (CALR), decorin (DCN), and PDGF-α at the mRNA level in *Borrelia*/fibroblast co-cultures. Intact *Borrelia* attach to and transmigrate fibroblasts, and undergo cystic transformation outside the fibroblasts. Fibroblasts preserve their vitality and express a prominent granular endoplasmic reticulum, suggesting activated protein synthesis. On two different semi-quantitative real-time PCR assays, BB- and BA/fibroblast co-cultures showed a significant induction of type I collagen mRNA after 2 days compared to fibroblasts (fourfold for BA and 1.8-fold for BB; *p* < 0.02). In addition, there was a significant upregulation of mRNA expression of TGF-β, CALR, PDGF-α, and DCN in BA and BB co-cultures compared to control fibroblasts in monolayer cultures after 2 days (*p* < 0.01). The BA/fibroblast co-culture induced a considerably greater upregulation of collagen and growth factor mRNA compared to BB/fibroblast co-culture. In contrast, a significant down-regulation of FGF-1 (20-fold for BA and 4.5-fold for BB) mRNA expression was detected in co-cultures compared to controls (*p* < 0.01). The results of the study support the hypothesis that BB sensu lato, and BA in particular, enhances collagen mRNA expression and can stimulate growth factors responsible for increased collagen production.

## Introduction

*Borrelia burgdorferi* sensu lato, a bacterium transmitted by ticks into the skin of humans and animals, has a high affinity for connective tissue and binds to decorin, fibronectin, integrin, plasminogen, glycosaminoglycans, and other components of connective tissue [9, 28, 33]. Borrelia may invade type I collagen fibres [52] and also fibroblasts, where they are protected from the action of antibiotics such as ceftriaxone [23].

Lyme borreliosis in Europe is caused by three main genospecies of *B. burgdorferi* sensu lato, namely, *B. burgdorferi* sensu stricto (BB), *B. afzelii* (BA), and *B. garinii* (BG) [29]. Only BB exists in the United States. A certain organotropism of the different genospecies has been observed. BA, the most common detected genospecies in Europe, has a high affinity for the skin. Its persistence causes the chronic infection known as acrodermatitis chronica atrophicans [10, 35]. Skin infection of long duration is characterized by atrophy on one hand and skin sclerosis on the other, as seen in ACA with pseudoscleroderma [3]. In the brain, reactive gliosis or astrogliosis evidenced by condensation of astrocytic fibres was observed in the rhesus macaque infected with a neurotropic *B. burgdorferi* strain [37]. Degenerative lesions and fibrosis were seen in peripheral nerves [38]. Experimental studies on monkeys have shown that BB infection of heart tissue also induces fibrosis, as shown by a significantly increased density score [7].

Fibrosis of the skin has been repeatedly observed in association with a *Borrelia* infection in Europe only, namely, circumscribed scleroderma (cSc), lichen sclerosus et atrophicus, exacerbation of systemic scleroderma, trigger finger, or the carpal tunnel syndrome [5, 6, 15, 40, 48]. Since sclerotic skin lesions have not been reported in the United States, the question arises as to whether the predominant genospecies in Europe, BA or BG, can influence and exaggerate collagen turnover. In cSc, the BA genospecies was isolated from skin biopsies and DNA of BA- and BG-, but not BB*-*DNA has been detected in cSc patients’ skin biopsies from patients in Germany, Austria, and Japan but not in the United States [6, 11].

CSc and SSc are caused by excessive deposits of type I collagen [25], manifested by more than a threefold elevation of collagen I mRNA levels in SSc skin compared to controls [17]. This phenomenon is promoted by several cytokines and growth factors. Isolated scleroderma fibroblasts produce elevated mRNA levels of type I collagen and transforming growth factor (TGF-ß) in culture [44]. Furthermore, platelet-derived growth factor (PDGF-α) [21], calreticulin (CALR) [27, 54], decorin (DCN) [46], and other molecules are thought to be involved in the increased collagen syntheses [45]. Basic fibroblast growth factor (FGF-1) induces collagen production by stimulating skin fibroblasts and connective tissue growth factor (CTGF) [8].

Based on these reports, we studied the behaviour of fibroblasts in co-culture with different *Borrelia* genospecies. We looked for a difference between collagen syntheses in co-culture compared to fibroblasts only, induced either by BB or by BA. Our aim was to simultaneously show the interaction of fibroblasts with *Borrelia* by electron microscopy and investigate the synthesis of mRNA collagen type I as well as the synthesis of different growth factors and molecules known to stimulate collagen production compared to fibroblasts only.

## Materials and methods

### Culture of Borrelia

Three strains of *B. burgdorferi*, BB strain B31 (gifted by Bettina Wilske of the Max von Pettenkofer Institute in Munich, Germany), and two strains of BA, isolated from skin biopsies of patients with erythema migrans at the department of dermatology, Medical University of Graz (ethical approval No. 181 99/00), were used. The two BA isolates were typed by RFLP analysis at the Institute of Hygiene and Microbiology, Medical University of Graz, by Doris Stünzner [42].

*Borrelia* were stored at − 70 °C, thawed at room temperature, and cultured in 8-ml Falcon tubes at 34 °C in BSK-H medium [36]. Cultures were monitored once a week in regard of growth, vitality, movement of *Borrelia*, and the purity of the medium by dark-field microscopy. *Borrelia* were counted in a Petroff-Hausser counting chamber. For subcultures, 100–1000 μl of *Borrelia* suspension was inoculated in fresh BSK-H medium. *Borrelia* were subcultured to a density of 10^8^ cells/microscopic field.

### Culture of fibroblasts

The FF2462 fibroblast cell line was isolated from human foreskin [provided by Dr. Meenhard Herlyn (The Wistar Institute, Philadelphia, PA, USA)] [49]. The cells were stored at − 70 °C, thawed in warm water, and dissolved in 8 ml DMEM (500 ml DMEM supplemented with 50 ml FCS und 10 ml glutamine). Fibroblasts were centrifuged at 1200 rpm for 5 min, and the pellet was dissolved in 4 ml DMEM and applied to culture tubes of 75 cm^2^. Ten millilitres of DMEM was then added and the cells were cultured at 37 °C with 5% CO_2_. After 4 days, the supernatant was replaced by 10 ml fresh DMEM and cultured for another 3 days. Subcultures were performed once a week to a cell count of 10^5^ cells.

### Co-culture of fibroblasts with *B. burgdorferi* for studying cell morphology by electron microscopy

Four parallel analyses were started for the co-culture experiments using the BB B31 and the BA 1 strain; the latter showed better growth than BA2. Cultures could be harvested after 24, 48, 72 h, and 7 days. For the experiments, a polypropylene lattice was placed in each well of a 6-well plate, and then, 10^5^ fibroblasts in 2 ml DMEM were pipetted in the well. After 24 h, DMEM was withdrawn and replaced by 1 ml RPMI. After centrifugation of *Borrelia* cultures at 1500 rpm, the pellet was resuspended in 1 ml of RPMI containing 10^8^ *Borrelia*/ml, added to the wells, and incubated for 24, 48 and 96 h, and 7 days, respectively.

For morphological analysis, the fluid of the plate was withdrawn and the cells were removed from the lattices, fixed for 30 min in 2.5% glutaraldehyde in 0.1 M cacodylate buffer pH 7.4 for 2 h, rinsed in cacodylate buffer for 2 h at 4 °C, and centrifuged at 800 rpm for 5 min. The pellet was placed in a 2% agarose solution, cooled to 4 °C, and then cut into small blocks which were again fixed in 2.5% glutaraldehyde in cacodylate buffer for 2 h. The specimens were dehydrated and embedded in TAAB embedding resin (TAAB, Aldermaston). They were sectioned at 60 nm using a Leica UCT ultramicrotome (Leica Microsystems, Vienna, Austria), stained with lead citrate and uranyl acetate, and analysed with a Zeiss EM 902 transmission electron microscope (Carl Zeiss Oberkochen).

### RNA isolation and semi-quantitive RT-qPCR

Total RNA was extracted using Trizol (Invitrogen, Carlsbad, CA, USA) according to the manufacturer’s instructions. cDNA was synthesized using the RevertAid^™^ H Minus first strand cDNA synthesis kit (Fermentas, St. Leon-Rot, Germany). To investigate the mRNA synthesis of collagen type I, two different primer pairs were used (col 1–1 fw: 5′-AAA CAA TGG TGC TCA GGG AC-3′ and col 1–1 rev: 5′-AGG ACC AGG GAG ACC AAA CT-3′; Col 1–2 fw: 5′-CAG CAC CTT CTC TCA GAC CC-3′ and Col 1–2 rev: 5′-GCA TCC TTG GTT AGG GTC AA-3′). The mRNA of FGF1, PDGF-α and TGF-β, CALR, and DCN were measured with a commercially available primer assay (Qiagen, Hilden, Germany).

Semi-quantitative real-time PCR (RT-qPCR) was performed in triplicate using an ABI Prism 7000 Detection System (Applied Biosystems, Carlsbad, CA, USA). Reaction mix (25 μl): 1 × SYBR^®^ Green PCR Master Mix (Invitrogen, Carlsbad, CA, USA), forward and reverse primer (1 mM each), 3 μl cDNA. Glyceraldehyde-3-phosphate dehydrogenase (GAPDH), and hypoxanthine–guanine phosphoribosyltransferase (HPRT1) served as housekeeping genes. The results are expressed as relative units based on calculation 2^− ΔΔCT^, which yields the relative amount of target gene normalised to the endogenous control (mean of two housekeeping genes) and relative to peripheral blood mononuclear cells. The cycling protocol was as follows: one cycle of 50 °C for 2 min and 95 °C for 10 min, followed by 50 cycles consisting of denaturation for 15 s at 95 °C, annealing of primers and elongation for 1 min at 60 °C.

### Statistical analysis

All statistical analyses were performed using the Statistical Package for Social Sciences, version 17.0 (IBM, NY, USA). Differences in expression levels were analysed using the Mann–Whitney *U* test. Spearman’s correlation test was performed to examine any correlation of collagen type I expression to FGF-1, PDGF-α, TGF-β, CALR, and DCN. A *p* value lower than 0.05 was considered to indicate statistical significance. Expression levels are presented as means ± standard deviation. All statistical tests were two-sided.

## Results

### *Borrelia* bind to fibroblasts, invade them, and undergo extracellular cystic transformation

**Fig. 1:**
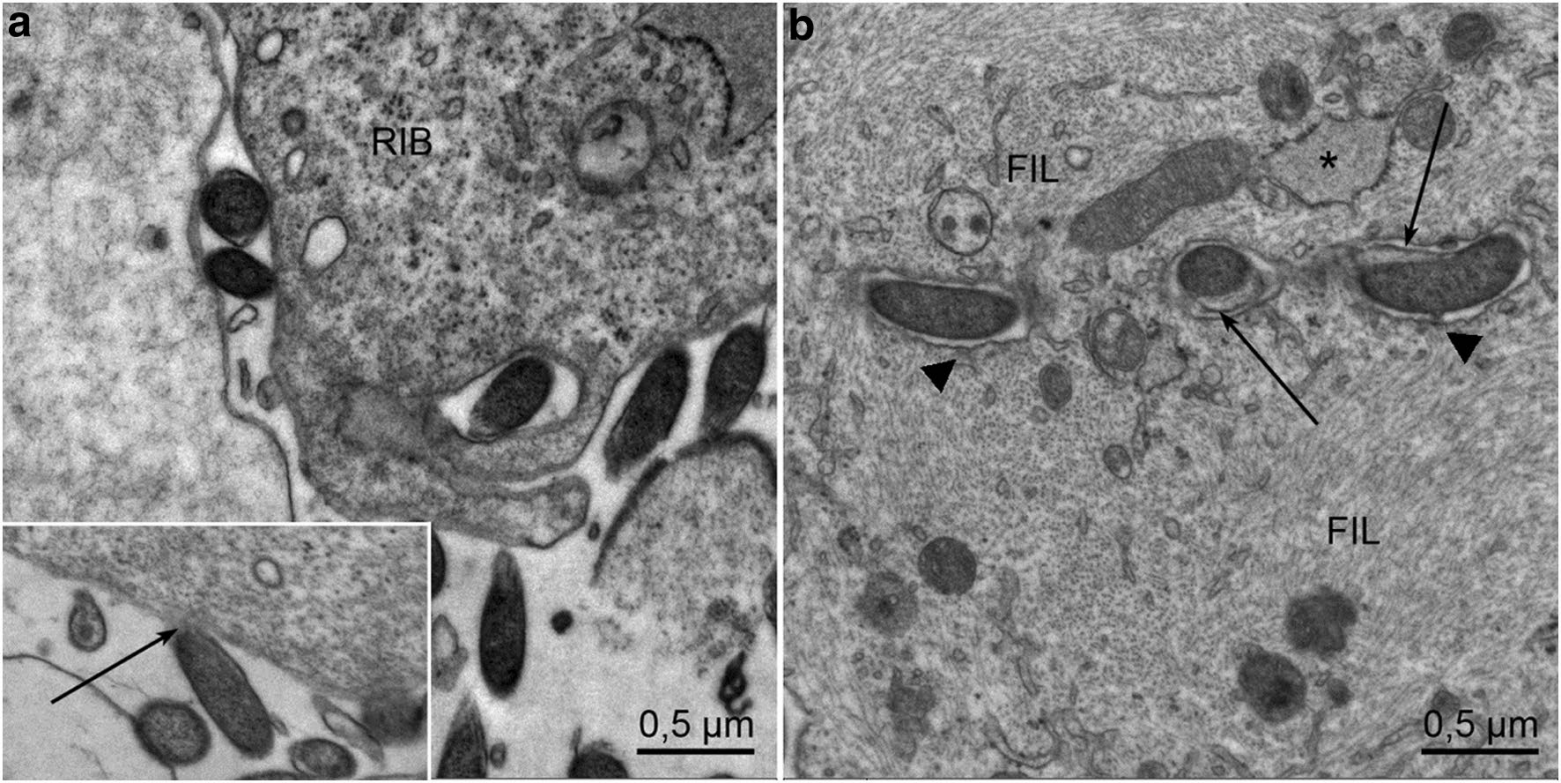
**a** Interaction of *B. afzelii* and fibroblasts after co-culture for 24 h: *B. afzelii* bind to fibroblast membranes by tethers shown by an arrow. *Borrelia* seem to be invaginated in fibroblasts. Both the membranes of *Borrelia* and fibroblasts are intact. Ribosomes (RIB). **b** After 48 h intact, *Borrelia* is enclosed in a vesicle in the fibroblast’s cytoplasm, thus indicating that *Borrelia* passes through the cytoplasm. Multiple parallel and cross-sectioned filaments labelled as FIL—probably actin microfilaments—are seen in the vicinity with condensations at the fibroblast membrane surrounding the *Borrelia* forming attachment plaques for the filaments marked by triangles. Outer envelope of *B. afzelii* is marked by arrows. Endoplasmic reticulum is labelled by an asterisk

Morphologic changes were identical when using BB B31 and BA in co-culture with fibroblasts in RPMI. Owing to better tissue preservation, the presented images are shown for the BA/fibroblast co-culture. The first changes were noted after 24 h of co-culture. BA bind to fibroblast membranes, forming tethers or being surrounded by a cytoplasmic extension (Fig. 1a). Even at this stage, some *Borrelia* seem to be enclosed in fibroblasts, thus preserving the membranes of both *Borrelia* and fibroblasts. Invagination and the passage of *Borrelia* through the fibroblast become evident after 48 h (Fig. 1b). *Borrelia* are surrounded by a dense microfilament network (diameter ~ 6 nm) showing condensations at the fibroblast`s plasma membrane (Fig. 1b).

**Fig. 2:**
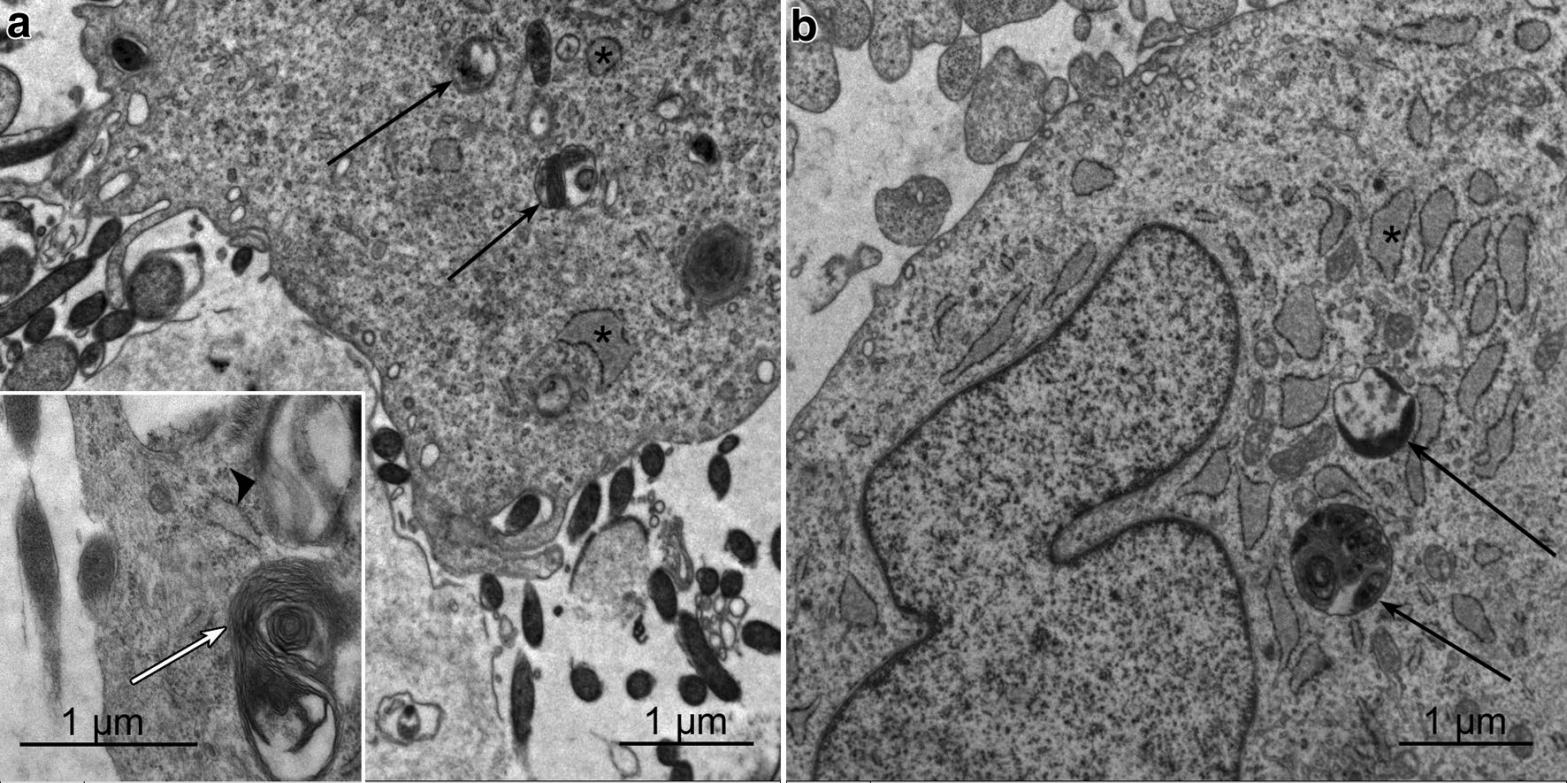
After 24 h co-culture with *B. afzelii* (**a**) and control fibroblasts (**b**) exhibit autophagolysosomes shown by arrows. Inset in **a**: putative degenerative form of *B. afzelii* enclosed in a vacuole marked by arrowhead and a myelin body labelled by an open arrow. Endoplasmic reticulum is labelled by an asterisk

**Fig. 3:**
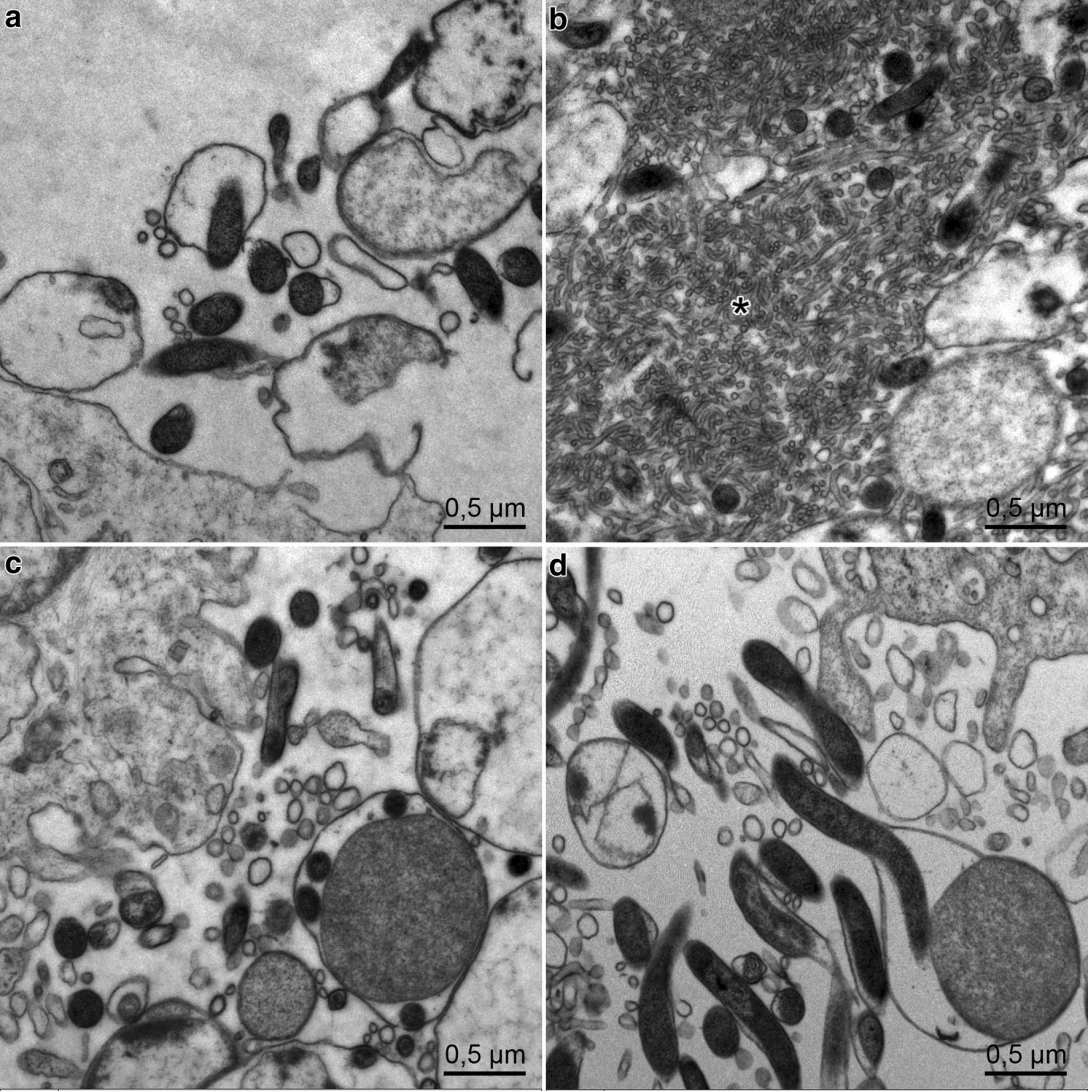
Extracellular changes of *B. afzelii*: cyst formation, shedding of the outer membrane with numerous outer membrane blebs and tubular structures marked by an asterisk **a** after 24 h; **b** after 48 h; **c, d** after 96 h

**Fig. 4:**
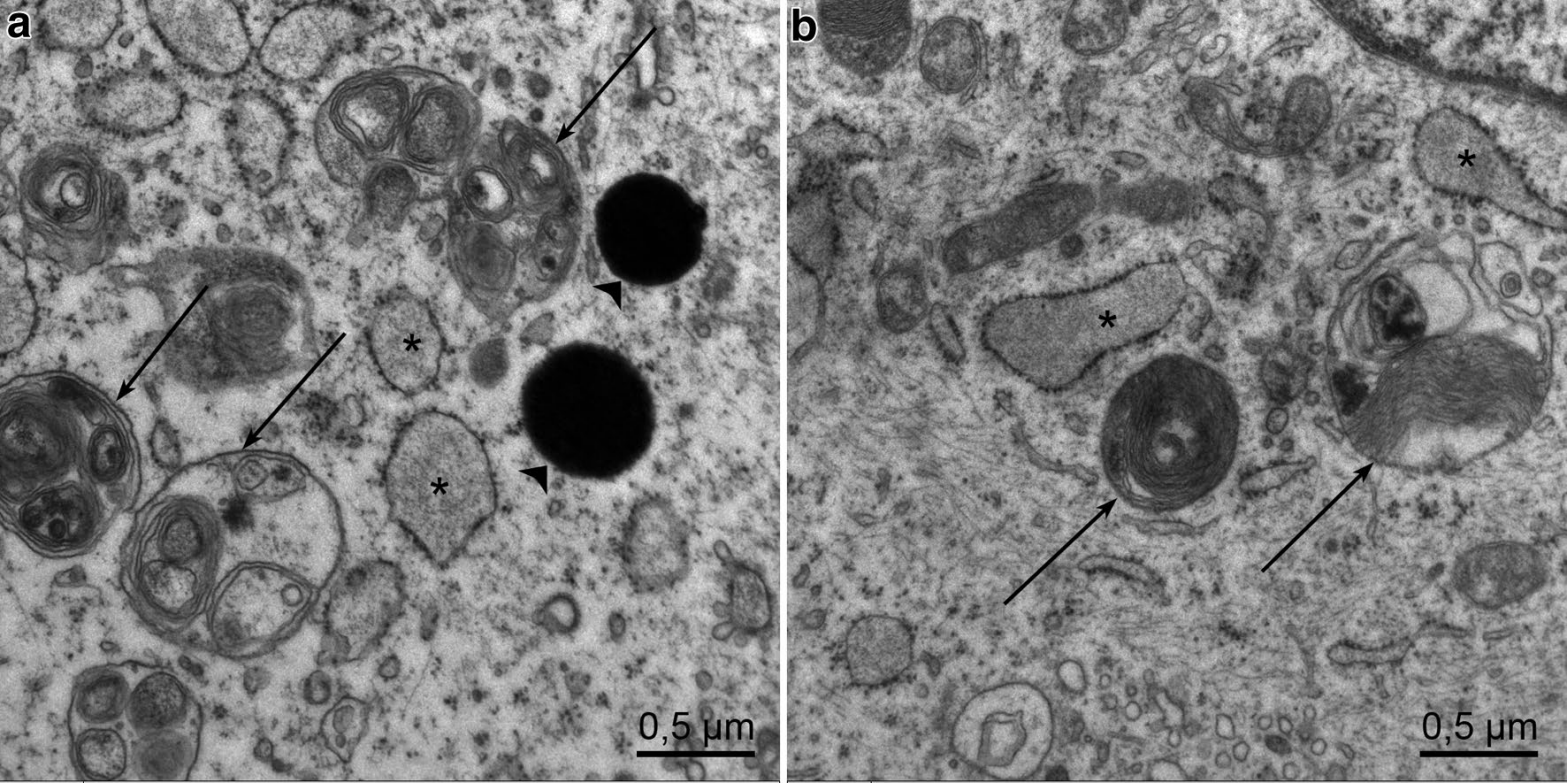
**a** Fibroblast co-culture with *B. afzelii* after 72 h. Numerous autophagolysosomes shown by arrows and lipid droplets shown by arrowheads. **b** Control fibroblast after 24 h of co-culture with autophagolysosomes of different shapes marked by arrows. Endoplasmic reticulum is labelled by an asterisk

At all timepoints and also after 72 h, the fibroblasts are intact, showing a prominent granulated endoplasmic reticulum (Figs. 1b, 2a, b, 4a, b). In contrast, *Borrelia* show enlargement of the outer membrane and some outer membrane blebs after 24 h (Fig. 3a), after 48 h abundant shedding of the outer membrane with multiple blebs and tubules in extracellular location (Fig. 3b). After 96 h, cyst and gemma formation of *Borrelia* is predominant (Fig. 3c, d). Degenerative changes were seen within the fibroblasts after 1 week, which hindered their assessment by electron microscopy. Fibroblasts of co-culture and control fibroblasts showed myelin bodies, lipid droplets, and autophagolysosomes, indicating that autophagic processes were taking place in the fibroblasts, both in co-cultures and in control cultures (Fig. 4a, b).

### Type I collagen mRNA expression is significantly increased in fibroblast/*B. burgdorferi* co-cultures compared to fibroblasts only

**Fig. 5:**
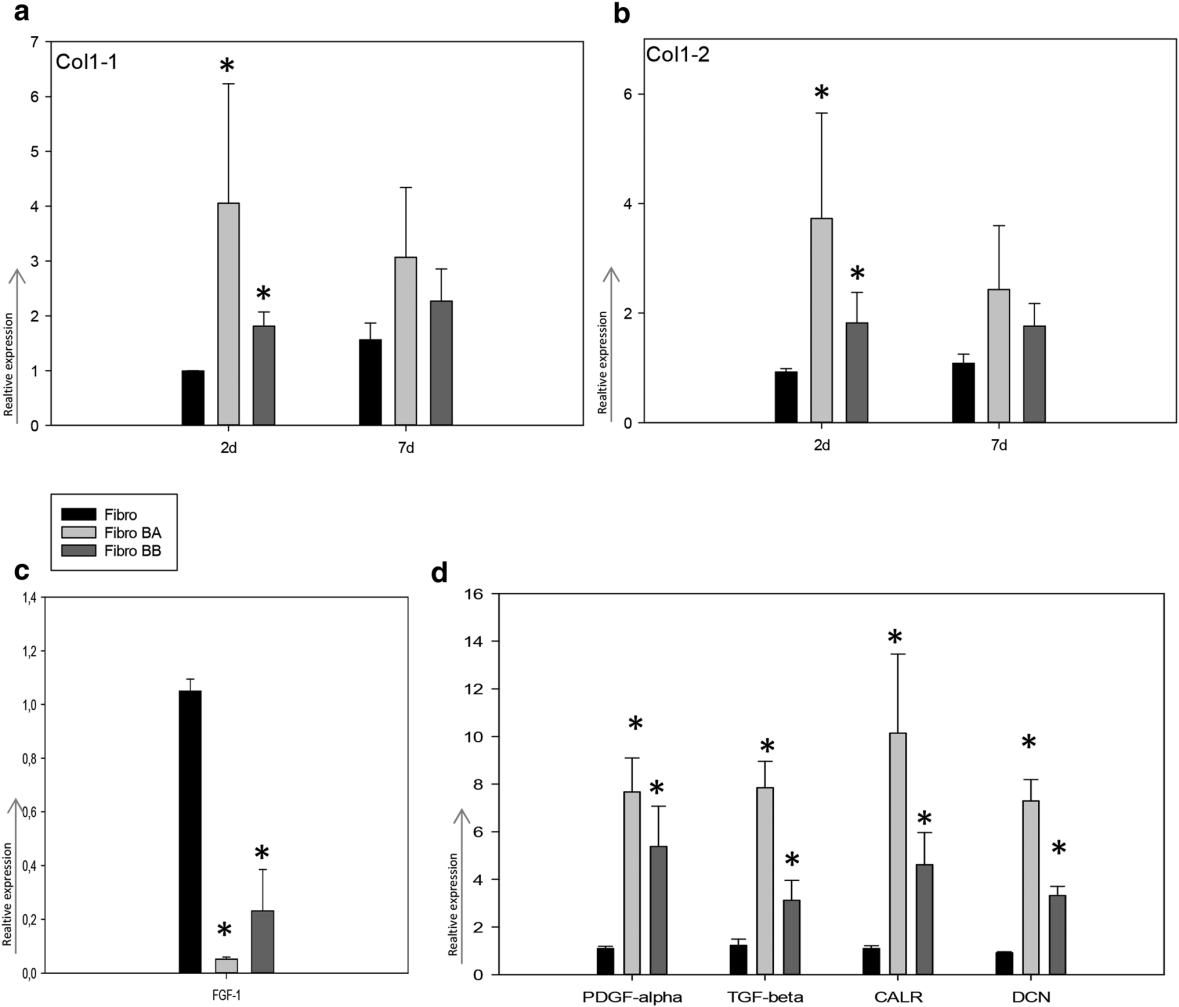
Type 1 collagen, FGF-1, PDGF-alpha, TGF beta, CALR and DCN expression in fibroblasts (Fibro), fibroblasts co-cultured with *B. afzelii* (Fibro BA) and fibroblasts co-cultured with *B. burgdorferi* sensu stricto B31 (Fibro BB). **a, b** Type 1 collagen expression of four technical replicates of the two PCR assays (Col1-1 and Col1-2). **c** FGF-1 expression of three biological replicates. **d** PDGF-alpha, TGF beta, CALR and DCN expression of three biological replicates. Each bar represents the mean expression of error bars indicate standard error of the mean. Stars indicate significant deregulation compared to controls

Using two different semi-quantitative real-time PCR assays, BA and BB co-cultures were associated with a significant mRNA induction of type I collagen after 2 days compared to cultured fibroblasts (around fourfold for BA and 1.8-fold for BB for both assays, *p* < 0.02), (Fig. 5a, b). No significant difference in type I collagen mRNA expression was detected after 7 days when comparing co-cultures with cultured fibroblasts.

### Growth factors that regulate collagen metabolism are significantly upregulated in fibroblast/*B. burgdorferi* co-cultures

mRNA expression analyses of growth factors demonstrated a significant upregulation of TGF-β (sixfold for BA and 2.5-fold for BB), CALR (ninefold for BA and 4.2-fold for BB), PDGF-α (sevenfold), and DCN (eightfold) in BA and BB/fibroblast co-cultures compared to control fibroblasts, after 2 days (*p* < 0.01) (Fig. 5d). In contrast, a significant down-regulation of FGF-1 mRNA expression (20-fold for BA and 4.5-fold for BB B31) was detected in BA and BB co-cultures compared to controls (*p* < 0.01) after 2 days (Fig. 5c). However, none of these growth factors revealed any significant difference between BA and BB co-cultures. Correlating type I collagen expression to mRNA levels of their regulating growth factors and proteins, a significant positive correlation of TGF-β (Spearman’s rho 0.728 *p* = 0.026), PDGF-α (Spearman’s rho 0.703 *p* = 0.035), CALR (Spearman’s rho 0.829 *p* = 0.006), and DCN (Spearman’s rho 0.795 *p* = 0.041) was observed. In addition, expression levels of FGF-1 were negatively correlated with type I collagen expression (Spearman’s rho − 0.705 *p* = 0.035).

## Discussion

In the present study, we registered, for the first time, the excessive stimulation of collagen mRNA synthesis in fibroblast cultures when co-cultured with *Borrelia*. Furthermore, mRNA expression of the growth factors TGF-β, PDGF-α, CALR, and DCN was significantly correlated with collagen mRNA synthesis.

In agreement with the previous findings, we noted that *Borrelia* enter fibroblasts by forming tethers between the cell wall of the *Borrelia* and the cell membrane of the fibroblast, then transmigrate through these cells, and are continuously surrounded by the fibroblast’s cell membrane. Klempner also studied co-cultures by electron microscopy and observed that *Borrelia* were internalized in a host vesicle [23]. Wu and co-workers reported that *B. burgdorferi* is able to internalise into different eukaryotic cells including endothelial cells, fibroblasts, neuronal, and neuroglial cells [51]. The entry of *Borrelia* was also studied in Vero cells [16]. At 24–48 h, most Vero cells had spirochetes either attached to their surface or internalized. Numerous coated pits and vesicles (120–150 nm diameter) were attached to the penetrating spirochetes. The uptake of *Borrelia* by fibroblasts differed in our study. *Borrelia* were attached to the cell surface or folded into the cell membrane of fibroblasts, as noted in synovial cells [12].

Fibronectin has been shown to mediate *Borrelia* attachment [9, 24]. However, the invasion of *Borrelia* requires β-1 integrins [51]. Fibrinogen binding by *Borrelia* may create a bridge for binding integrins [18]. The decorin-binding protein A is important for tethering, as observed on endothelial cells. Furthermore, P66, a porin with adhesive capability, may support the transmigration of *Borrelia* and intracellular invasion [18]. The cytoskeleton is responsible for internalisation of bacteria, as observed in a *Bacillus anthracis*/fibroblasts cell line [39]. Wu et al. suggested that the reorganisation of actin filaments and Src family kinases is necessary for internalisation [51]. We found densely packed filaments in close apposition to Borrelia within fibroblasts, probably representing actin filaments.

It has been reported that the internalisation of *B. burgdorferi* does not alter the viability of mammalian cells [51]. The viability of fibroblasts was similar in both, co-cultures and control cultures of fibroblasts alone; after 1 week autophagolysosomes were found in both cultures.

*Borrelia* are known to undergo pathologic changes such as shedding of the outer envelope and cystic malformation under adverse conditions like exposure to penicillin or deprivation of nutrients in the culture fluid [22, 51]. In our study as well, *Borrelia* showed typical bleb and gemma formation, and cystic expansion as a sign of malnutrition. The latter might have been induced by co-culture in RPMI and deprivation of important nutrients for the growth of *Borrelia*, as is true of the BSK-H medium. We co-cultured fibroblasts with *Borrelia* according to a previous protocol by Klempner et al., which suggests RPMI as culture medium [23].

Both *Borrelia* species BB and BA are associated with similar morphologic changes. However, the role of the genospecies-specific immune response has not been studied in detail. In the recent past, human dermal fibroblasts co-cultured with the three main *B. burgdorferi* sensu lato species showed a homogenous inflammatory gene profile with similar transcriptional profiles and no species-specific fingerprint of transcriptional changes in fibroblasts, including a common core of chemokines/cytokines and interferon-related genes [30].

We found a considerable upregulation of TGF-β mRNA synthesis in fibroblasts co-cultured with BA, resulting in a fourfold increase of collagen type I mRNA and a 1.8-fold increase after exposure to BB compared to control fibroblasts.

The distribution of collagens I and III in the skin varies in the different skin layers with substantial differences in expression of mRNA for type I and III procollagen [4]. In morphea, fibroblast cell lines produced increased amounts of type I and type III collagens, but the ratios of type I and type III collagens remained relatively unchanged in all the cultures, suggesting that they have undergone a coordinated activation of collagen synthesis at transcriptional level [47].

We only investigated type I collagen mRNA synthesis in our co-culture experiments, because type I collagen is the predominant type in normal and sclerotic skin [2]. Human fibroblasts in culture synthesize both type I and type III collagen, with type I accounting for 70–90% of the total. The proportion of type III collagen is dependent on culture conditions and differs in patients with certain connective tissue diseases. Whereas the proportion of type III collagen in normal fibroblast cell lines is increased at high cell density, cells from patients with systemic sclerosis grow at a lower density than control cells and have a reduced type III/I ratio. It could well be that fibroblasts exposed to *B. burgdorferi* produce type III collagen in a different way we did not look for.

TGF-β is known to markedly enhance the production of collagen types I and III. In human dermal fibroblasts exposed to TGF-β, Varga et al. observed a two to threefold increase in the quantities of collagen measured by hydroxy [^14^C] proline synthesis [43]. Total RNA extracted from skin biopsies of systemic sclerosis showed a greater than threefold elevation of collagen I mRNA levels compared to controls [17]. TGF-β is a key regulator of extracellular matrix assembly and remodelling. Elevated TGF-β expression in the affected organs correlates with connective tissue deposits in the process of fibrosis [45]. TGF-β 2 mRNA was found to be co-localised with collagen I expression in patients with the inflammatory stage of systemic sclerosis [26]. Similarly, decorin works together with TGF-β in controlling fibrosis [14].

Collagen turnover is regulated by local growth factors that stimulate fibroblasts to produce extracellular matrix proteins. Chaperones play an important role in maintaining the quality of protein processing in the endoplasmic reticulum (ER) [13]. In our study, co-cultured and control fibroblasts were rich in granular ER, indicating active RNA synthesis [31]. Calreticulin is an ER chaperone upregulated by ER stress in fibrotic tissue [54]. CALR regulates type I collagen transcription and processing into the extracellular matrix (ECM). Fibroblasts with increased CALR expression enhance the response to TGF-ß. A correlation has been reported between CALR upregulation and the progression of fibrosis. CALR was also upregulated in a bleomycin-induced animal model of lung fibrosis [27]. In our *Borrelia*/fibroblast co-culture, the production of CALR was significantly elevated at the mRNA level, thus enhancing collagen synthesis.

DCN is a key regulatory molecule in collagen fibril assembly [53]. The role of DCN in scleroderma was studied by Vuorio et al. Fibroblasts were cultured from affected skin areas of patients with systemic and localised scleroderma, and investigated in regard of mRNA levels of TGF-ß and DCN. Whereas TGF-ß mRNA was found to be slightly elevated in fibroblasts, DCN mRNA showed marked variations in cell lines and was not correlated with collagen mRNA [46]. In our study, DCN mRNA was significantly overexpressed in *Borrelia*/fibroblast co-cultures compared to fibroblast cultures. BB expresses DNC binding proteins at the outer surface; these are lipoproteins which bind decorin and glycosaminoglycans [41]. In laboratory mice with chronic infection, *Borrelia* were found to absolutely co-localise with decorin but not with collagen I [20]. In an autopsy study, *B. burgdorferi* was found in the cardiac interstitium of five patients who experienced sudden cardiac death. *B. burgdorferi* was associated with collagen fibres and co-localised with DCN as well, as shown by immunohistochemistry [32].

PDGF is involved in fibrosis associated with systemic sclerosis. PDGF receptors are expressed on collagen-secreting fibroblasts [21]. Resident dermal fibroblasts and/or myofibroblasts promote skin fibrosis by overproducing the collagen-rich extracellular matrix. PDGF-α mRNA was significantly higher in *Borrelia*/fibroblast co-cultures than in control fibroblasts.

Compared to the action of different growth factors inducing increased collagen RNA synthesis, FGF-1 had the opposite effect. A significant down-regulation of FGF-1 mRNA expression in co-cultures was found to be negatively correlated with collagen I expression. This confirmed previous data reported by Ichiki et al., who showed that FGF is a potent inhibitor of basal and TGF-β-stimulated collagen expression in human skin fibroblasts [19].

To our knowledge, the role of different growth factors in collagen synthesis has not yet been investigated in LB. One of the major limitations of the present study was that no data were available for secreted growth factors at the protein level. The results reported here must be validated in further experiments. We also do not know whether autocrine secretion of growth factors in fibroblasts can influence fibroblast activity and increased collagen gene expression, such as with bFGF, PDGF, or ascorbic acid which is a constituent of the culture medium [1, 34, 50]. Because these factors are part of the media, we suppose that they are working together with paracrine and autocrine stimulation of fibroblasts. Besides, cytokines or growth factors produced by the interaction of *Borrelia* with the immune system may further influence collagen gene expression which, to our knowledge, has not yet been studied in LB.

Our findings support the hypothesis that skin fibrosis can be triggered by *Borrelia*, presumably BA and to a lesser extent also BB B31, by stimulating several profibrotic molecules such as TGF-β, CALR, DCN, PDGF-α. All of these, with the exception of FGF, are positively correlated with mRNA collagen expression. The difference in the levels of collagen mRNA which were considerably but not significantly higher in BA co-culture compared to BB may suggest a higher risk for fibrosis in countries with predominance of* B. afzelii*. Further strains of* B. burgdorferi* sensu lato should be co-cultured with fibroblasts, also of other tissues like tendon, and fibroblast cultures from sclerotic lesions of circumscribed scleroderma. Whether these findings may have implications for clinical entities such as circumscribed scleroderma, lichen sclerosus et atrophicus, carpal tunnel syndrome, or the trigger finger, remains to be seen. The exact mechanism of action in vivo is unknown and will have to be studied in further in vitro studies and animal models.
